# Suspected Hematuria: Adverse Effects of Rivaroxaban in Older Adult Treated for Atrial Fibrillation

**DOI:** 10.3390/reports7010011

**Published:** 2024-02-08

**Authors:** Aleksandra Rapaić, Ekaterina Milošević, Nemanja Todorović, Nataša Janjić, Mladena Lalić-Popović, Nataša Milošević

**Affiliations:** 1 AU Biofarm, 21000 Novi Sad, Serbia; bacvanin.agi@gmail.com; 2 Department of Pharmacy, Faculty of Medicine, University of Novi Sad, 21000 Novi Sad, Serbia; ekaterinamilosevic1234@gmail.com (E.M.); nemanja.todorovic@mf.uns.ac.rs (N.T.); mladena.lalic-popovic@mf.uns.ac.rs (M.L.-P.); 3 Department of Orthopaedic Surgery and Traumatology, Clinical Centre of Vojvodina, Faculty of Medicine, University of Novi Sad, 21000 Novi Sad, Serbia; natasa.janjic@mf.uns.ac.rs

**Keywords:** adverse drug reaction, rivaroxaban, omeprazole, ciprofloxacin, pharmaceutical healthcare

## Abstract

Background: The modern concept of pharmaceutical healthcare implies monitoring the pharmacotherapy outcomes and reporting adverse drug reactions. Objective: To present a suspected hematuria as the adverse rivaroxaban reaction in a patient with atrial fibrillation observed by pharmacists in a community pharmacy. Case presentation: A 69-year-old female patient came to a pharmacy with a prescription for cranberry-based supplement. She was diagnosed with a mild urinary infection after experiencing blood in her urine for about two weeks. The pharmaceutical anamnesis revealed that the patient was treated with irbesartan and rivaroxaban. Rivaroxaban was applied for atrial fibrillation, and the patient was treated for nine months. The patient was treated with omeprazole gastro-resistant capsules for mild dyspepsia and stomach ache over a three-week period. The pharmacist counselled the patient to contact the clinician who introduced rivaroxaban, further suggesting substitution with different anticoagulant. Although the urine culture was negative, the physician introduced ciprofloxacin, which was followed by blood in the patient’s stool. Thus, gastroscopy, colonoscopy, and gynecological examination were advised. All findings were normal. Four days after rivaroxaban was substituted with acenocoumarol, no blood in the urine or stool was detected. Conclusions: Rivaroxaban can cause spot urine blood even when applied in therapeutic doses among older female patients when applied with omeprazole. Possible rivaroxaban interaction with omeprazole metabolites is suspected and should be carefully monitored.

## 1. Introduction

Medication-related errors cost approximately USD 42 billion a year globally [1]. Medication error is any failure that causes or which may cause harm to patients when medications are applied. Errors may be due to inappropriate or ineffective prescribing, including over- and under-prescribing, as well as a result of a mistake in writing the prescription, dispensing, and/or administering it. Some medication errors, as a result, lead to an adverse drug reaction. Adverse drug reaction is defined as “an appreciably harmful or unpleasant reaction, resulting from an intervention related to the use of a medicinal product, which predicts hazard from future administration and warrants prevention or specific treatment, or alteration of the dosage regimen, or withdrawal of the product” [2], whereas an adverse event is “any abnormal sign, symptom or laboratory test, or any syndromic combination of such abnormalities, any untoward or unplanned occurrence (e.g., an accident or unplanned pregnancy), or any unexpected deterioration in a concurrent illness”. Adverse events which can be attributed to medication are recognized as suspected adverse drug reactions. Not every adverse event is a result of a drug application, although they do occur while a patient is taking a drug. Adverse events are not exclusively reactions to a medicine but can be attributed to a failure of certain standards. In addition, adverse drug reactions may be the result of medication error or may be expected due to a drug’s mechanism of action [3,4]. All health workers (clinicians, physicians, nurses, and pharmacists) involved in the treatment process who deal with medicines or medical products should follow certain standards and procedures in order to prevent failure. 

Aging is one of the most significant global problems affecting the world’s healthcare and social services systems. Projections indicate that by 2050, the number of elderly (aged 65 and over) people will reach 38 percent of the world’s population [5]. The prevalence of multimorbidity among elderly people is estimated to be from 55 to 98% [6], and this is followed by polymedication, which dramatically increases the risk of medication-related problems. It is estimated that home-dwelling older adults with multiple chronic conditions visit the emergency department due to medication-related problems in as much as 25% of total cases [7].

Pharmacists provide pharmaceutical care, which includes comprehensive work with a patient from initial evaluation of therapy to identifying drug-related problems and counseling [8,9]. The pharmaceutical care concept allows collaborative action with other health workers involved in the treatment process. The implementation of pharmaceutical care services prevent adverse drug reactions, drug-related morbidity, and hospitalization, but it also improves life quality, which results in an expenses reduction of up to USD 5377 per avoided adverse event. The early integration of a clinical pharmacologist in the patient treatment and therapy review significantly reduced both the risk of fatal outcome and hospitalization duration [10]. Pharmacists have both the knowledge and skills needed to improve patients’ medication use [9,11].

The Good Apothecary Guidance of the Pharmaceutical Chamber of Serbia recognizes the importance of therapy review and monitoring the therapy outcome and gives standards and recommendations [9] for conducting this service in both hospital and community pharmacy. The standards and recommendations are in accordance with Medication Therapy Management defined by the American Pharmacy Association (APhA) and include [12,13,14] the following:

- Performing a comprehensive medication review of prescribed drugs, nonprescription drugs (over-the-counter, OTC medication), dietary products, and medical devices for each indication;
- Selecting, initiating, modifying, or administering medication therapy;
- Providing verbal education and training designed to enhance patient understanding of the type and purpose of the prescribed drugs, OTC medication, dietary products, and medical devices, as well as education about the recommended dosage regimen, the time of administration, and, if so applicable, specific requirements (for example, taking medication at a specific time, taking medication with a meal or after fasting, the appropriate application of inhalation dosage forms, etc.), and other important recommendations;
- Providing information, support services, and resources designed to enhance patient adherence, such as education about the risks of irregular medication intake or the self-initiated discontinuation of therapy, as well as the monitoring of problems and/or difficulties related to therapy application;
- Monitoring and evaluating the safety of applied therapy, such as identifying, resolving, and preventing medication-related problems, including adverse drug events by controlling the risk of a cumulative effect caused by certain drugs and evaluating the significant contraindications, possible drug–drug, drug–food, or drug–disease/condition interactions, and/or adverse effects;
- Monitoring and evaluating patients’ response to therapy and effectiveness of the applied therapy, namely the control of the desired therapeutic outcomes;
- Documenting the pharmaceutical care provided for the patient and the pharmaceutical intervention that was undertaken, as well as communication with other healthcare providers.

In accordance with all the abovementioned assessments, a pharmacist implements appropriate patient counseling and education, and, if necessary, refers the patient to the physician/clinician or informs them about the recognized need for optimizing the patient’s therapy [9,12]. 

In this paper, a pharmaceutical care service, which has helped to recognize a drug-related problem, is presented. 

## 2. Detailed Case Description

A 69-year-old female patient (BMI 24.2 kg/m^2^) came to a community pharmacy under a urologist’s recommendation to buy a cranberry-based OTC preparation for the treatment of a mild urinary infection. Through a conversation with the pharmacist, it was understood that she had experienced painless hematuria for about two weeks. The causes of gross hematuria include traumatic injury, neoplasm, urinary tract infection, pyelonephritis, glomerulonephritis, radiation cystitis, and metabolic, congenital, hematologic, vascular, obstructive, and idiopathic causes. Nontreated hematuria may lead to kidney failure [15]. After the pharmacist reviewed her therapy, it was noted that the patient was treated with Irbenida^®^ (irbesartan) 150 mg 1 × 1, Xarelto^®^ (rivaroxaban) 20 mg 1 × 1, and Omeprol^®^ (omeprazole) caps. 1 × 1. The patient was diagnosed nine months ago with atrial fibrillation, and she was treated with Xarelto^®^ (rivaroxaban) 20 mg 1 × 1 over that period. During the last three weeks, she was introduced to Omeprol^®^ (omeprazole) caps. 1 × 1 for mild dyspepsia and stomach ache by her physician. The patient was administered Omeprol^®^ (omeprazole) regularly. The blood in her urine appeared about two weeks ago; meanwhile, the patient visited her physician, where she subsequently undertook a urine test. The test showed no specific changes, so she was prescribed cranberry-based OTC preparation. Cranberry-based preparation should not be applied in patients who are treated with anticoagulant therapy, specifically warfarin [16]. However, there is not sufficient evidence of rivaroxaban interactions with cranberry; given the fact that the patient was already experiencing hematuria, cranberry supplementation would not be the best choice. Namely, cranberry juice in vitro is proven to be an inhibitor of CYP enzymes, to be as potent as ketoconazole (CYP3A) and fluconazole (CYP2C9) in higher amounts [17]. The main pathway of rivaroxaban metabolism is mainly through CYP3A4 [18], and thus it may interfere additionally to rivaroxaban, causing potential adverse effects. Since the urine test did not show any infectious cause of hematuria, she was advised to contact her physician and address the internal medicine specialist who introduced Xarelto^®^ tbl.20 mg into her therapy. The patient was informed that the appearance of blood in her urine is a common side effect of this drug. The patient visited the community pharmacy the next day after being examined by her physician. An antibiotic therapy Ciprocinal^®^ (ciprofloxacin) tbl. 500 mg 2 × 1 for 10 days was introduced by the physician despite sterile urine results. Moreover, she was appointed for a consultation with a gynecologist upon the suggestion of her physician. After being examined by the gynecologist and no pathological findings were reported, the gynecologist agreed with the pharmacist’s assumption that the blood in the urine could be an adverse effect of rivaroxaban.

**Figure 1:**
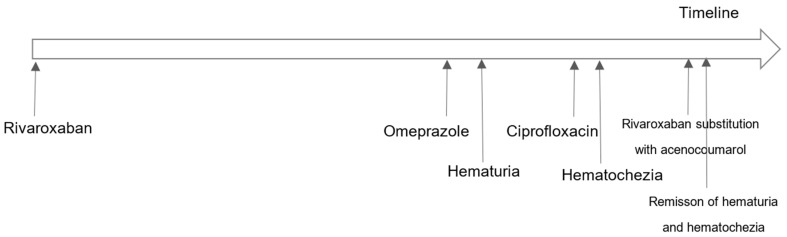
Timeline of the adverse events and their remission.

In the meantime, after blood appeared in her stool, the patient addressed her physician at the health center, who insisted on finishing the antibiotic therapy before further analyses were conducted. Afterwards, the patient voluntarily reported to a specialist doctor who included Xarelto^®^ tbl. Into her therapy of atrial fibrillation. The internal medicine specialist asked for the gastroscopy and colonoscopy findings before any change in the therapy was conducted. The gastroenterological findings indicated no pathological changes, and thus Xarelto^®^ tbl. 20 mg was discontinued and Acenocoumarol UNION^®^ (acenocoumarol) tbl. 4 mg was introduced. Only four days later, the patient visited the pharmacy to report that there was no visible blood neither in her urine or stool, and that she subjectively felt better and stronger. The timeline of these events is summarized in Figure 1.

The Naranjo algorithm, or adverse drug reaction probability scale [19], was also applied to assess whether there was a causal relationship between hematuria and rivaroxaban using a simple questionnaire to assign probability scores. The score obtained by the Naranjo algorithm is given in Table 1, with a total score of 5. 

The Naranjo scale has intervals spanning from −4 to +9. A total score from 5 to 8 indicates probable adverse drug reaction. According to the interpretation of the Naranjo scale result, the adverse reaction is recognized as probable since it (i) followed a reasonable temporal sequence after rivaroxaban was applied, (ii) followed a recognized response, namely hematuria to the suspected drug, i.e., rivaroxaban, (iii) was confirmed by the withdrawal of rivaroxaban, and (iv) could not be reasonably explained by the known characteristics of the patient’s clinical state since urinary infection was never confirmed. The suspected occurrence of an adverse drug reaction was reported to the Medicines and Medical Devices Agency of Serbia through their website.

## 3. Discussion

In the presented case, rivaroxaban was introduced in a patient with nonvalvular atrial fibrillation in order to reduce the risk of stroke and systemic embolism. The antithrombotic therapy recommendation for all patients with increased risk of stroke after being identified with nonvalvular atrial fibrillation is in accordance with the opinions of the experts of the American College of Chest Physicians (ACCP), American Stroke Association (ASA), and American Heart Association (AHA). The antithrombotic therapy choice is based on the balance between the patient’s risk of stroke on the one side and the risk of bleeding on the other side. In general, patients with a moderate to high risk of stroke (prior ischemic stroke or TIA, advanced age ≥75 years, hypertension, diabetes mellitus type 2, or congestive heart failure) and acceptably low risk of bleeding are considered for oral anticoagulant therapy. Patients at low risk of stroke are recommended to apply acetyl-salicylic acid or no antithrombotic therapy. Anticoagulant therapy is more than justified in this case because female patients in advanced age with diagnosed hypertension and atrial fibrillation are at high risk of stroke [20,21,22]. Rivaroxaban is no less effective than warfarin in stroke prevention among patients with nonvalvular atrial fibrillation [23]. The risk of hemorrhage increases with rivaroxaban introduction to therapy, and older patients are more prone to experience thrombotic and bleeding events. It is described that rivaroxaban can cause serious, sometimes fatal bleeding. Regardless of age, the risk-to-benefit rivaroxaban profile is satisfactory [20,21,22]. Nevertheless, rivaroxaban is superior to warfarin in the prevention of venous thromboembolism according to the newest data [24]. Moreover, rivaroxaban and other direct oral anticoagulants (DOACs) have a more rapid and predictable anticoagulant response, and the need for routine laboratory monitoring is significantly reduced in comparison to warfarin [20,21,22,24].

After oral application, rivaroxaban is almost completely absorbed (80–100%) under both fed and fast conditions, with 2–4 h required for the drug to reach maximum plasma concentrations. Rivaroxaban binds reversible to plasma proteins mainly to albumin up to 92–95%. Approximately one-third of the applied drug is being excreted unchanged through renal excretion, while two-thirds of the dose undergoes metabolic biotransformation. It is estimated that approximately 18% of rivaroxaban’s elimination can be attributed to CYP3A4, while CYP2J2 participates with 14% of total rivaroxaban clearance. The produced metabolites are excreted through renal and hepatobiliary pathways [18].

Age-related pharmacokinetics changes in rivaroxaban have been reported. Among elders (over 75 years), increased rivaroxaban exposure (increased AUC and elevated plasma concentrations) has been observed in comparison to younger (18–45 years) volunteers. Rivaroxaban exposure in advanced age may be elevated up to 50%, probably due to diminished both renal and nonrenal clearance. No sex-related alterations in the pharmacokinetics of rivaroxaban have been reported [25]. Moreover, rivaroxaban plasma concentrations among subjects with body weight extremes (≤50 or >120 kg) had nonsignificant changes when compared to those in volunteers with average body weight (70–80 kg) [26].

The enhanced anticoagulant effect of rivaroxaban in the observed patient could not be attributed to obesity. But, the patient is a 69-year-old female, so her age may be taken as a considerable factor that affects the clearance rate of rivaroxaban, which has led to probable increased drug exposure. Although no drug interactions of rivaroxaban with irbesartan, omeprazole, or ciprofloxacin have been reported to the best of our knowledge, possible combination of mature age and even weak drug–drug interactions should be taken under careful consideration.

**Figure 2:**
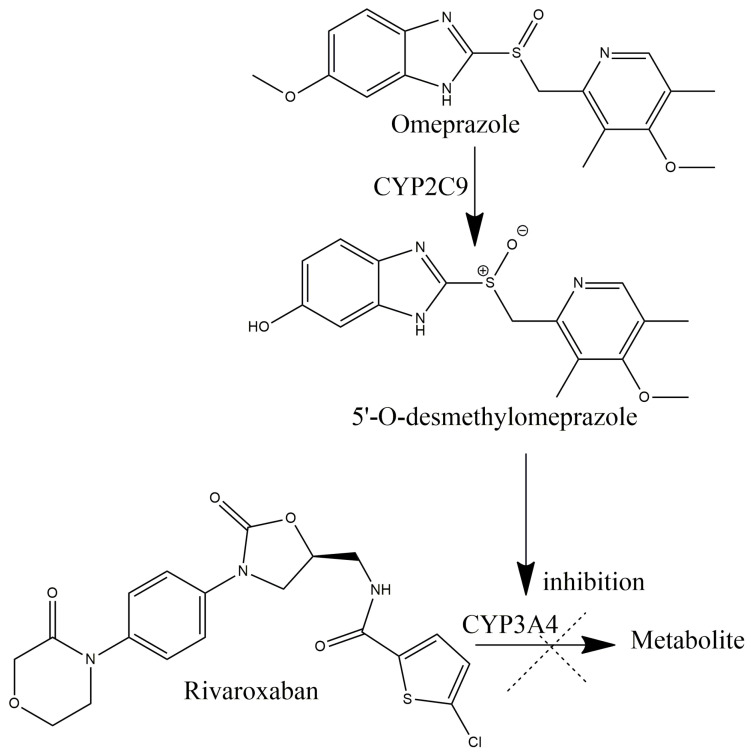
Possible mechanism of omeprazole–rivaroxaban interaction.

Irbesartan is an angiotensin receptor blocker indicated in the therapy of hypertension and diabetic nephropathy with a long duration of action. It is usually introduced in therapy once daily. The metabolism of irbesartan combines two metabolic phases: oxidation in phase I and glucuronidation in phase II. The dominant pathway of irbesartan biotransformation exhibits through CYP2C9 activity, while the metabolism via CYP3A4 is considered to be negligible. In the second phase of metabolism, irbesartan undergoes glucuronidation by UGT1A3. Thus, no interaction with rivaroxaban can be expected [27]. Omeprazole, a proton pump inhibitor, is highly metabolized by CYP enzymes. The majority of its biotransformation occurs through CYP2C19 action, whereas 5-hydroxyomeprazole (OH-OMP) and 5′-O-desmethylomeprazole (DM-OMP) are produced, while the remaining dose of the drug is converted into omeprazole sulfone (OMP-S) by CYP3A4 activity [28]. Omeprazole is a reversible in vivo [29,30] and in vitro [31,32] inhibitor of both CYP2C19 and CYP3A4. However, a recent study of the omeprazole metabolites contribution towards the inhibition of CYP activity sheds light on their importance in omeprazole–drug interactions. Based on experimental data, DM-OMP, which is a product of CYP2C19 activity, was demonstrated to be responsible for the majority of CYP3A4 inhibitions [33]. Even though it has been labeled that omeprazole does not affect rivaroxaban pharmacokinetics, omeprazole metabolites may have a substantial role in its interaction with drugs metabolized dominantly through CYP3A4 (Figure 2). 

Medication-related problems, including adverse drug reactions, medication errors, and nonadherence, are more often observed and reported among polymedicated older adults [34]. In addition, older adults are more prone to drug–drug interactions when polymedicated. Almost 50% of community-dwelling elderly adults had a potential drug–drug interaction, and the risk of interaction increased with the number of medications [35]. Medication-related problems, especially drug–drug interactions and adverse drug effects, are preventable when approached with appropriated care. Therapy review and management may considerably decrease the number of medication-related hospital admissions, rehospitalizations, institutionalization in convalescent hospitals and nursing homes, and premature death, especially among the elderly [34,35]. Unrecognized drug–drug interactions have a significant contribution towards incorrect DOACs dosing; consequently, there is justified suspicion that adverse DOACs effects can be attributed to concomitant application with other drugs. Nevertheless, rivaroxaban has fewer drug–food and drug–drug interactions in comparison to warfarin. The key points for rivaroxaban interactions with other medicines or food are mediated by the cytochrome P450 enzyme and/or the transporter permeability glycoprotein (P-gp). It has been reported that amiodarone as a CYP3A4 and P-gp inhibitor elevates rivaroxaban plasma concentration, with a higher risk of clinically significant adverse effects of rivaroxaban in patients with impaired renal function. Increased rivaroxaban drug levels are observed in patients with reduced kidney function who are treated with diltiazem, a moderate CYP3A4 inhibitor, and P-gp substrates. The concomitant application of rivaroxaban with P-gp and strong CYP3A4 inducers such as rifampin, carbamazepine, phenytoin, and phenobarbital should be avoided according to the recommendations listed on the FDA label [36,37].

Ciprofloxacin, which was introduced for the treatment of possible urine infection, is metabolized through CYP1A2 and could not be suspected for possible metabolic interactions with existing therapy. However, ciprofloxacin may affect the gut flora, leading to disbyosis and reduced vitamin K production by the microbiota. Although no interactions between ciprofloxacin and rivaroxaban have been reported yet, to the best of the authors’ knowledge, the concomitant application of ciprofloxacin and warfarin is associated with increased risk of bleeding, and few cases in susceptible patients have been reported [38,39].

In this case, blood in the urine was detected before ciprofloxacin was introduced into therapy; actually, ciprofloxacin was applied due to blood spots in the patient’s urine samples. One should note that after the patient started to use ciprofloxacin, aggravation of the existing coagulopathy was observed, and blood appeared not only in the urine but also in the stool. However, the symptoms of the patient did not alleviate after she finished the antibiotic therapy. It requires time for the gut microbiota to renew after antibiotics therapy; thus, the effects of rivaroxaban exposure may be prolonged. In addition to this presumption, the patient was not treated with probiotics during the antibiotic treatment.

## 4. Conclusions

The pharmacist suspected blood in the urine and later in the stool to be an adverse rivaroxaban effect rather than a new symptom or a sign of an undiagnosed condition. At this point, due to limited data, it is speculative to make final conclusions whether the rivaroxaban-enhanced effect is a result of ciprofloxacin–gut microflora dysbiosis–rivaroxaban association, or whether it is attributed to prolonged omeprazole metabolites–rivaroxaban interactions. Nevertheless, therapy review and patient counseling as a part of pharmaceutical care practice and as a pharmacist’s activity in community pharmacy is pivotal to recognize adverse drug effects and drug–drug interactions.

## Figures and Tables

**Table 1:** The score of adverse drug reaction probability scale.

Question	Yes	No	Do Not Know	Score
1. Are there previous conclusive reports on this reaction?	+1	0	0	0
2. Did the adverse event appear after the suspected drug was administered?	+2	−1	0	+2
3. Did the adverse event improve when the drug was discontinued or a specific antagonist was administered?	+1	0	0	+1
4. Did the adverse event reappear when the drug was readministered?	+2	−1	0	0
5. Are there alternative causes that could on their own have caused the reaction?	−1	+2	0	+2
6. Did the reaction reappear when a placebo was given?	−1	+1	0	0
7. Was the drug detected in blood or other fluids in concentrations known to be toxic?	+1	0	0	0
8. Was the reaction more severe when the dose was increased or less severe when the dose was decreased?	+1	0	0	0
9. Did the patient have a similar reaction to the same or similar drugs in any previous exposure?	+1	0	0	0
10. Was the adverse event confirmed by any objective evidence?	+1	0	0	0
	Total Score 5			

## Data Availability

The data presented in this study are available on request from the corresponding author. The data are not publicly available due to privacy.
